# Supplementary material for: Using the president’s tweets to understand political diversion in the age of social media
Source: Nat Commun. 2020 Nov 10;11:5764. doi: 10.1038/s41467-020-19644-6 (PMC7655817; doi:10.1038/s41467-020-19644-6)
Supplement: Supplementary file 1 — Supplementary Information [file 41467_2020_19644_MOESM1_ESM.pdf]

Supplementary Material for:
Using the president's tweets to understand political diversion in the age of social media

Stephan Lewandowsky, Michael Jetter, Ullrich K. H. Ecker

Summary statistics for the content items

Table S1 provides summary statistics for all content items, namely the president's tweets, NYT coverage, and ABC News headlines. The variable Missing days refers to days on which no tweets were posted on @realDonaldTrump or for which no archival coverage was available for ABC News.

Autocorrelation structure

We first established the autocorrelation structure for each of the variables under consideration by regressing the observations on day t onto a varying number of lagged observations of the dependent variable from days $t-1$, $t-2$, ..., $t-k$. All models also included fixed effects for each week of the sample, as well as linear and quadratic time trends, for a total of $N = 106$ additional control variables. Tables S2 through S5 show the results for the tweets with presumed diversionary items, the *New York Times*, and ABC News, respectively. The 5 numbered columns in each table show different models with a different number (k) of lagged variables. In each table, the bold-faced model was chosen for the analyses reported in the paper.

Sensitivity analysis for targeted analysis

The results of the sensitivity analysis for the average-media model in Table 1 (columns 3 and 6) are shown in Table S6 and Figure S1. The crucial entries in Table S6 are the robustness values (RV), which identify the amount of residual variance of both the treatment and the outcome that an omitted variable would have to explain in order to explain away the estimated treatment effect (which is summarized by the first four entries in the table). Specifically, for the diversion model, an unobserved confounder would have to explain 13.3% of the residual variance for the effect of media coverage to be zero ($RV_{q=1}$), and would have to explain 6.1% for the effect to be no longer significant at $\alpha = .05$ ($RV_{q=1,\alpha=.05}$). Both of those values are greater than the strength of association observed for the largest lag effect included in the model (lag 4; row labeled "Bound (1 $\times$ lags)"). For suppression, the situation is similar, although the difference between the values of RV and the bounds provided by the largest lag effect (lag 5) is considerably smaller.

The likelihood of an omitted variable explaining away the effects of our predictors can be further assessed through Figure S1. In each panel, the dashed red line represents the significance threshold for the diversion coefficient (left-hand panel) and the suppression coefficient (right-hand panel). Points below and to the left of that dashed line correspond to a region where potential unobserved confounders would not affect result. We can populate that space by taking an included variable from the model—in this case the lag with the largest value of t from the set of lags included in each model—and show the effects of an unknown confounder relative to the size of the largest lag effect. The three red diamonds in each panel refer to a confounder having an association with the outcome that is equal to, twice as great, or three times as large (i.e., "1/2/3 $\times$ lags" in Figure S1) as the known role of the largest lag.

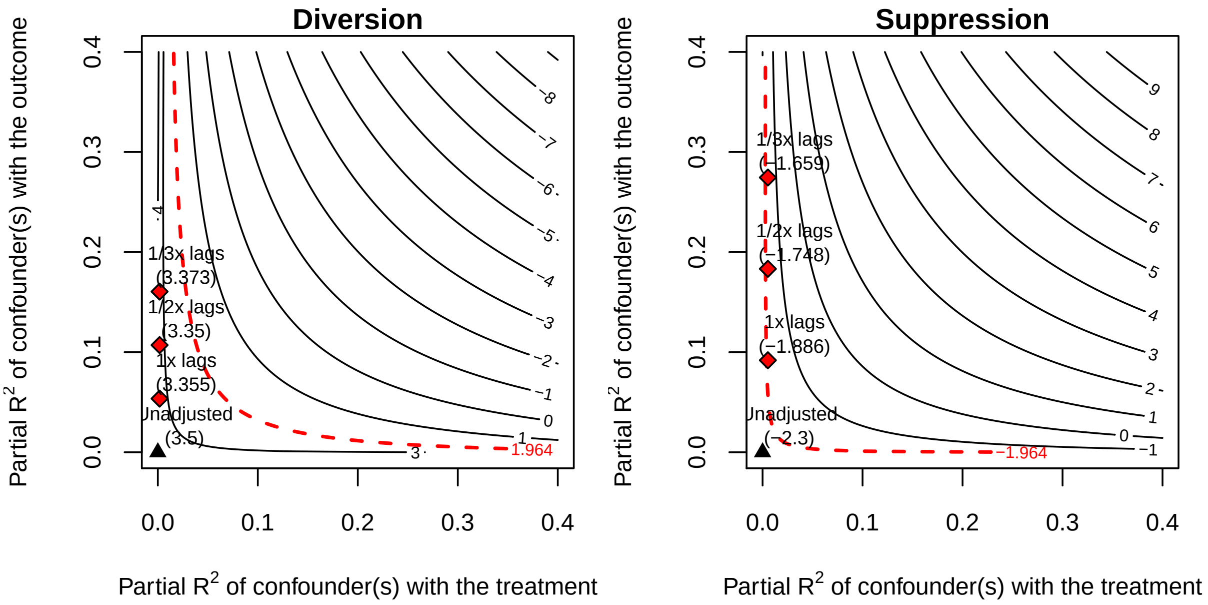

Figure S1: Sensitivity contour plots with benchmark bounds. The dashed red line in each panel represents the significance threshold for the t-value of the coefficient of interest (for diversion in the left-hand panel and suppression on the right). The region below and to the left of the dashed line envelopes values of a potential unobserved confounder that would not affect significance of the result. The red diamonds in each panel represent hypothetical values of an omitted confounder whose association with the outcome is equal in strength to the largest t-value of lags represented in the model (point labeled $1\times$lags), or twice as strong ($2\times$lags) or three times as strong ($3\times$lags). The reference lag was lag 4 for diversion and lag 5 for suppression.

It is clear that for the diversion model, an omitted variable would have to have an association with the treatment far greater than the most powerful lag for this unknown confounder to explain away the diversion effect. For the suppression model, by contrast, an omitted variable with equal associative strength to the largest lag effect (point "(1 $\times$ lags)" in Figure S1), would (just) render the coefficient non-significant ($p \simeq .06$). If the confounder were three times as strong (point "(3 $\times$ lags)"), the significance of the suppression coefficient would be $p \simeq .10$.

Taken together, the sensitivity analysis lends support to our hypothesis that adverse media coverage causes the president to engage in diversion, and that this diversion, in turn, causes the media to reduce that coverage. Quantification of the likelihood that an omitted confounder is instead responsible for the effect shows that endogeneity is considerably less likely to be a concern for the diversion model than the suppression model.

Disentangling keywords used in targeted analysis

The targeted analysis reported in Table 1 in the main text considered all three critical keywords ("China", "jobs", and "immigration") together. Tables S7 through S12 present the same analysis but broken down into individual keywords and pairs of keywords. For example, Table S7 presents the analysis for the keywords "China" and "jobs" (CJ), Table S12 presents the analysis for the single keyword "immigration", and so on.

Semantic analysis for the individual keywords used in the targeted analysis

Figures S2 through S4 provide wordclouds for the content of Trump's tweets that are selected on the basis of a single keyword. Each word cloud shows the 50 most frequent words contained in the selected tweets (after elimination of stopwords and function words and so on).

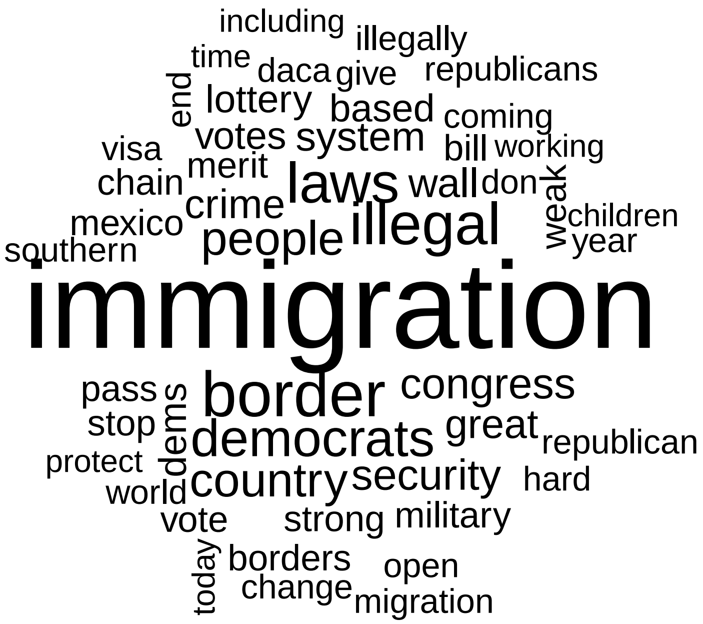

Figure S2: Word cloud representation of the top 50 words used in Donald Trump's tweets containing the keyword "immigration". Size of each word corresponds to its frequency, as does font color. The darker the font, the greater the frequency.

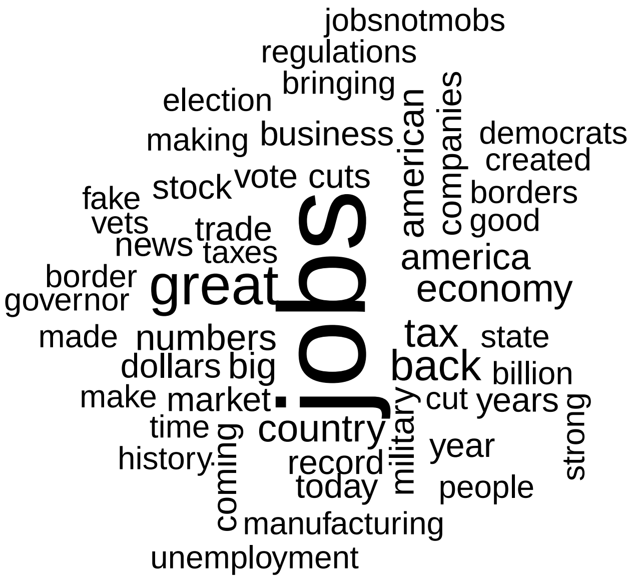

Figure S3: Word cloud representation of the top 50 words used in Donald Trump's tweets containing the keyword "jobs". Size of each word corresponds to its frequency, as does font color. The darker the font, the greater the frequency.

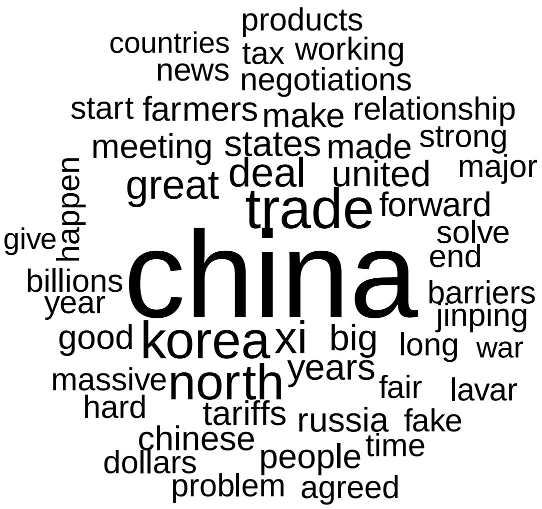

Figure S4: Word cloud representation of the top 50 words used in Donald Trump's tweets containing the keyword "China". Size of each word corresponds to its frequency, as does font color. The darker the font, the greater the frequency.

Negative binomial regression model for targeted analysis

Because the dependent variables in our analyses were event counts, the preferred analysis should involve a model for counted data. In particular, a negative-binomial regression is often considered to be the most appropriate model for analysis. Table S13 reports a negative binomial analysis of the main result in our targeted analysis, involving the 3 Twitter keywords "China", "jobs", and "immigration." The analysis used the *glm.nb* function in the R package *MASS*.

Negative binomial regressions are known to suffer from frequent convergence problems, and the present analysis was no exception, with the suppression model for ABC failing to converge. Because the negative binomial model also could not be applied to the average data, the paper therefore focuses on the OLS results which do not suffer from these problems.

Summary of word pairs identified in expanded analysis

Table S14 summarizes the number of word pairs observed in the critical bottom-right quadrants of Figure 4 (ie., tweets that occurred significantly more often in response to Russia/Mueller coverage in the media and that were associated with successful suppression of coverage the next day). Table S15 identifies these word pairs in decreasing order of effect size, defined to be the average of the two (absolute) t-values from the two independent OLS models. In line with the targeted analysis, the word "job" (or "jobs") was present in 49 out of the 62 word pairs, and it featured in virtually all of the top 10 pairs.

Table S1: Summary statistics for content items during the sampling period 20 January 2017 through 20 January 2019.

Source	N	Missing days	Mean	SD	Min	Max
@realDonaldTrump	5,040	12	7.01	4.03	1	24
New York Times	115,298	0	157.73	52.74	50	282
ABC News	7,214	40	10.44	1.85	5	15

Table S2: Exploring lag structure for Trump tweets on "China", "jobs", "immigration" (CJI).

Model:	(1)	(2)	(3)	**(4)**[a]	(5)
	$k = 1$	$k = 2$	$k = 7$	$k = 10$	$k = 14$
Dependent variable: CJI tweets on day t					
CJI tweets$_{t-1}$	-0.063 (0.050)	-0.066 (0.050)	-0.146 (0.050) ***	-0.158 (0.050) ***	-0.164 (0.050) ***
CJI tweets$_{t-2}$		-0.050 (0.060)	-0.110 (0.050) **	-0.119 (0.050) **	-0.120 (0.050) **
CJI tweets$_{t-3}$			-0.197 (0.060) ***	-0.219 (0.060) ***	-0.224 (0.060) ***
CJI tweets$_{t-4}$			-0.202 (0.050) ***	-0.232 (0.050) ***	-0.240 (0.050) ***
CJI tweets$_{t-5}$			-0.183 (0.050) ***	-0.224 (0.050) ***	-0.237 (0.050) ***
CJI tweets$_{t-6}$			-0.137 (0.050) ***	-0.176 (0.050) ***	-0.194 (0.050) ***
CJI tweets$_{t-7}$			0.001 (0.050)	-0.027 (0.050)	-0.045 (0.050)
CJI tweets$_{t-8}$				-0.088 (0.040) *	-0.110 (0.040) **
CJI tweets$_{t-9}$				-0.126 (0.040) ***	-0.148 (0.040) ***
CJI tweets$_{t-10}$				-0.058 (0.040)	-0.083 (0.040) *
CJI tweets$_{t-11}$					-0.035 (0.040)
CJI tweets$_{t-12}$					-0.046 (0.040)
CJI tweets$_{t-13}$					-0.036 (0.040)
CJI tweets$_{t-14}$					-0.069 (0.040) *
N	730	729	724	721	717

Notes: [a] Bold-faced model was chosen for the analyses reported in paper.

Table S3: Exploring lag structure for NYT Russia/Mueller coverage.

Model:	(1)	(2)	(3)	(4)	**(5)**^{a}
	$k = 1$	$k = 7$	$k = 14$	$k = 21$	$k = 28$
Dependent variable: NYT articles on Russia/Mueller on day t					
NYT Rus/Muell$_{t-1}$	0.312 (0.040) ***	0.076 (0.040) *	0.056 (0.040)	0.024 (0.050)	-0.012 (0.050)
NYT Rus/Muell$_{t-2}$		-0.062 (0.050)	-0.064 (0.050)	-0.091 (0.040) **	-0.098 (0.040) **
NYT Rus/Muell$_{t-3}$		-0.189 (0.040) ***	-0.207 (0.040) ***	-0.228 (0.040) ***	-0.219 (0.040) ***
NYT Rus/Muell$_{t-4}$		-0.143 (0.040) ***	-0.132 (0.040) ***	-0.152 (0.040) ***	-0.152 (0.040) ***
NYT Rus/Muell$_{t-5}$		-0.307 (0.040) ***	-0.307 (0.040) ***	-0.311 (0.040) ***	-0.306 (0.040) ***
NYT Rus/Muell$_{t-6}$		-0.027 (0.040)	-0.095 (0.040) **	-0.142 (0.040) ***	-0.187 (0.040) ***
NYT Rus/Muell$_{t-7}$		0.198 (0.040) ***	0.094 (0.040) **	-0.005 (0.050)	-0.069 (0.050)
NYT Rus/Muell$_{t-8}$			-0.181 (0.040) ***	-0.211 (0.040) ***	-0.255 (0.040) ***
NYT Rus/Muell$_{t-9}$			-0.120 (0.040) ***	-0.148 (0.040) ***	-0.188 (0.040) ***
NYT Rus/Muell$_{t-10}$			-0.119 (0.040) ***	-0.156 (0.040) ***	-0.189 (0.040) ***
NYT Rus/Muell$_{t-11}$			-0.108 (0.040) ***	-0.140 (0.040) ***	-0.157 (0.040) ***
NYT Rus/Muell$_{t-12}$			-0.098 (0.040) ***	-0.132 (0.040) ***	-0.166 (0.040) ***
NYT Rus/Muell$_{t-13}$			-0.069 (0.040) *	-0.130 (0.040) ***	-0.193 (0.040) ***
NYT Rus/Muell$_{t-14}$			0.175 (0.040) ***	0.081 (0.040) **	0.005 (0.040)
NYT Rus/Muell$_{t-15}$				-0.115 (0.040) ***	-0.156 (0.040) ***
NYT Rus/Muell$_{t-16}$				-0.152 (0.040) ***	-0.187 (0.040) ***
NYT Rus/Muell$_{t-17}$				-0.096 (0.040) **	-0.141 (0.040) ***
NYT Rus/Muell$_{t-18}$				-0.101 (0.040) ***	-0.126 (0.040) ***
NYT Rus/Muell$_{t-19}$				-0.099 (0.040) ***	-0.144 (0.040) ***
NYT Rus/Muell$_{t-20}$				-0.064 (0.040) *	-0.136 (0.040) ***
NYT Rus/Muell$_{t-21}$				0.147 (0.040) ***	0.063 (0.040)
NYT Rus/Muell$_{t-22}$					-0.081 (0.040) **
NYT Rus/Muell$_{t-23}$					-0.070 (0.040) *
NYT Rus/Muell$_{t-24}$					-0.136 (0.040) ***
NYT Rus/Muell$_{t-25}$					-0.124 (0.040) ***
NYT Rus/Muell$_{t-26}$					-0.124 (0.040) ***
NYT Rus/Muell$_{t-27}$					0.002 (0.040)
NYT Rus/Muell$_{t-28}$					0.071 (0.040) *
N	730	724	717	710	703

Notes: ^{a} Bold-faced model was chosen for the analyses reported in paper.

Table S4: Exploring lag structure for ABC Russia/Mueller coverage.

Model:	(1)	(2)	(3)	(4)	**(5)**[a]
	$k = 1$	$k = 7$	$k = 10$	$k = 14$	$k = 21$
Dependent variable: ABC articles on Russia/Mueller on day t					
ABC Rus/Muell$_{t-1}$	-0.072 (0.050)	-0.164 (0.050) ***	-0.182 (0.050) ***	-0.185 (0.050) ***	-0.200 (0.050) ***
ABC Rus/Muell$_{t-2}$		-0.165 (0.050) ***	-0.185 (0.050) ***	-0.183 (0.050) ***	-0.199 (0.050) ***
ABC Rus/Muell$_{t-3}$		-0.190 (0.040) ***	-0.225 (0.040) ***	-0.230 (0.040) ***	-0.246 (0.040) ***
ABC Rus/Muell$_{t-4}$		-0.165 (0.040) ***	-0.202 (0.050) ***	-0.206 (0.050) ***	-0.222 (0.050) ***
ABC Rus/Muell$_{t-5}$		-0.218 (0.040) ***	-0.257 (0.050) ***	-0.265 (0.050) ***	-0.278 (0.050) ***
ABC Rus/Muell$_{t-6}$		-0.107 (0.040) **	-0.140 (0.040) ***	-0.152 (0.040) ***	-0.166 (0.050) ***
ABC Rus/Muell$_{t-7}$		-0.066 (0.040)	-0.102 (0.040) **	-0.115 (0.040) **	-0.140 (0.050) ***
ABC Rus/Muell$_{t-8}$			-0.129 (0.050) ***	-0.156 (0.050) ***	-0.182 (0.050) ***
ABC Rus/Muell$_{t-9}$			-0.073 (0.050)	-0.097 (0.050) **	-0.132 (0.050) **
ABC Rus/Muell$_{t-10}$			-0.060 (0.050)	-0.081 (0.050)	-0.128 (0.050) **
ABC Rus/Muell$_{t-11}$				-0.021 (0.050)	-0.059 (0.050)
ABC Rus/Muell$_{t-12}$				-0.009 (0.040)	-0.054 (0.050)
ABC Rus/Muell$_{t-13}$				-0.086 (0.040) **	-0.133 (0.040) ***
ABC Rus/Muell$_{t-14}$				-0.038 (0.040)	-0.086 (0.050) *
ABC Rus/Muell$_{t-15}$					-0.119 (0.050) **
ABC Rus/Muell$_{t-16}$					-0.039 (0.050)
ABC Rus/Muell$_{t-17}$					-0.066 (0.040)
ABC Rus/Muell$_{t-18}$					-0.097 (0.040) **
ABC Rus/Muell$_{t-19}$					-0.020 (0.050)
ABC Rus/Muell$_{t-20}$					0.008 (0.040)
ABC Rus/Muell$_{t-21}$					0.057 (0.040)
N	730	724	721	717	710

Notes: [a] Bold-faced model was chosen for the analyses reported in paper.

Table S5: Exploring lag structure for Average (NYT & ABC) Russia/Mueller coverage.

Model:	(1)	(2)	(3)	(4)	**(5)**[a]
	$k = 1$	$k = 7$	$k = 14$	$k = 21$	$k = 28$
Dependent variable: Average[b] articles on Russia/Mueller on day t					
Average Rus/Muell$_{t-1}$	0.143 (0.040) ***	-0.003 (0.040)	-0.020 (0.040)	-0.054 (0.040)	-0.092 (0.050) **
Average Rus/Muell$_{t-2}$		-0.028 (0.040)	-0.046 (0.050)	-0.090 (0.050) *	-0.109 (0.050) **
Average Rus/Muell$_{t-3}$		-0.162 (0.040) ***	-0.194 (0.040) ***	-0.201 (0.040) ***	-0.201 (0.040) ***
Average Rus/Muell$_{t-4}$		-0.164 (0.040) ***	-0.183 (0.040) ***	-0.181 (0.040) ***	-0.178 (0.040) ***
Average Rus/Muell$_{t-5}$		-0.275 (0.040) ***	-0.298 (0.040) ***	-0.300 (0.040) ***	-0.290 (0.040) ***
Average Rus/Muell$_{t-6}$		-0.077 (0.040) **	-0.111 (0.040) ***	-0.140 (0.040) ***	-0.163 (0.040) ***
Average Rus/Muell$_{t-7}$		0.086 (0.040) **	0.032 (0.040)	-0.015 (0.040)	-0.063 (0.040)
Average Rus/Muell$_{t-8}$			-0.125 (0.040) ***	-0.149 (0.040) ***	-0.196 (0.040) ***
Average Rus/Muell$_{t-9}$			-0.075 (0.040) *	-0.118 (0.040) ***	-0.159 (0.050) ***
Average Rus/Muell$_{t-10}$			-0.084 (0.040) **	-0.118 (0.040) ***	-0.147 (0.040) ***
Average Rus/Muell$_{t-11}$			-0.067 (0.040)	-0.073 (0.040)	-0.104 (0.040) **
Average Rus/Muell$_{t-12}$			-0.069 (0.040) *	-0.100 (0.040) **	-0.121 (0.040) ***
Average Rus/Muell$_{t-13}$			-0.070 (0.040) *	-0.122 (0.040) ***	-0.164 (0.040) ***
Average Rus/Muell$_{t-14}$			0.058 (0.040)	-0.010 (0.040)	-0.059 (0.040)
Average Rus/Muell$_{t-15}$				-0.098 (0.040) **	-0.141 (0.050) ***
Average Rus/Muell$_{t-16}$				-0.056 (0.050)	-0.109 (0.050) **
Average Rus/Muell$_{t-17}$				-0.082 (0.040) **	-0.124 (0.040) ***
Average Rus/Muell$_{t-18}$				-0.154 (0.040) ***	-0.194 (0.040) ***
Average Rus/Muell$_{t-19}$				-0.060 (0.040)	-0.107 (0.050) **
Average Rus/Muell$_{t-20}$				-0.030 (0.040)	-0.084 (0.040) **
Average Rus/Muell$_{t-21}$				0.134 (0.040) ***	0.050 (0.040)
Average Rus/Muell$_{t-22}$					-0.052 (0.040)
Average Rus/Muell$_{t-23}$					-0.097 (0.040) **
Average Rus/Muell$_{t-24}$					-0.132 (0.040) ***
Average Rus/Muell$_{t-25}$					-0.083 (0.040) **
Average Rus/Muell$_{t-26}$					-0.135 (0.040) ***
Average Rus/Muell$_{t-27}$					-0.041 (0.040)
Average Rus/Muell$_{t-28}$					0.082 (0.040) **
N	730	724	717	710	703

Notes: [a] Bold-faced model was chosen for the analyses reported in paper.

[b] Average of standardized counts (z-transformed) for NYT and ABC.

Table S6: Sensitivity analysis of the OLS regressions for average media coverage from columns (3) and (6) of Table 1 in main body.

Panel A: Diversion model

Outcome: Distracting tweets						
Treatment:	Est.	S.E.	t-value	$R^2_{Y\sim D\|\mathbf{X}}$	$RV_{q=1}$	$RV_{q=1,\alpha=0.05}$
Average media	0.226	0.064	3.507	2%	13.3%	6.1%
df = 605		*Bound (1x lags)*: $R^2_{Y\sim Z\|\mathbf{X},D}$ = 5.4%, $R^2_{D\sim Z\|\mathbf{X}}$ = 0.2%				

Panel B: Suppression model

Outcome: Average media coverage						
Treatment:	Est.	S.E.	t-value	$R^2_{Y\sim D\|\mathbf{X}}$	$RV_{q=1}$	$RV_{q=1,\alpha=0.05}$
Distracting Tweets	-0.05	0.021	-2.332	0.9%	9.3%	1.5%
df = 571		*Bound (1x lags)*: $R^2_{Y\sim Z\|\mathbf{X},D}$ = 9.2%, $R^2_{D\sim Z\|\mathbf{X}}$ = 0.5%				

Table S7: Predicting diversionary tweets (“China”, “jobs”; CJ) from threatening media coverage (Russia-Mueller) and predicting suppression from diversionary tweets.

Dependent variable:		CJ tweets$_t$		NYT^a	ABC^a	Average^b
	(1)	(2)	(3)	(4)	(5)	(6)
NYT Russia/Mueller$_t$	0.007					
	(0.004) $p = .058$					
	[0.004] $p = .053$					
ABC Russia/Mueller$_t$		0.203				
		(0.077) $p = .008$				
		[0.069] $p = .003$				
Average Russia/Mueller$_t$			0.180			
			(0.060) $p = .003$			
			[0.053] $p = .001$			
CJ tweets$_{t-1}$	^{o}	^{o}	^{o}	-0.495	-0.051	-0.067
				(0.221) $p = .025$	(0.022) $p = .019$	(0.022) $p = .002$
				[0.210] $p = .019$	[0.022] $p = .020$	[0.020] $p = .001$
N	721	721	721	703	710	703

Notes.

Each model included control variables for each week during the sampling period and long-term time trends as well as the appropriate number of lagged observations for the dependent variable (see Methods). Table entries are coefficients (OLS standard errors) [Newey-West standard errors].

^{a} Coverage of Russia-Mueller on day t.

^{b} Average of the standardized values of coverage of NYT and ABC.

^{o} Lagged predictor is shown only when of interest.

Table S8: Predicting diversionary tweets (“Jobs”, “immigration”; JI) from threatening media coverage (Russia-Mueller) and predicting suppression from diversionary tweets.

Dependent variable:	JI tweets$_t$			NYT^a	ABC^a	Average^b
	(1)	(2)	(3)	(4)	(5)	(6)
NYT Russia/Mueller$_t$	0.011					
	(0.004) $p = .007$					
	[0.004] $p = .009$					
ABC Russia/Mueller$_t$		0.184				
		(0.074) $p = .014$				
		[0.070] $p = .009$				
Average Russia/Mueller$_t$			0.195			
			(0.061) $p = .001$			
			[0.058] $p = .001$			
JI tweets$_{t-1}$	^{o}	^{o}	^{o}	-0.277	-0.030	-0.037
				(0.267) $p = .300$	(0.024) $p = .216$	(0.027) $p = .172$
				[0.291] $p = .342$	[0.022] $p = .186$	[0.027] $p = .167$
N	721	721	721	703	710	703

Notes.

Each model included control variables for each week during the sampling period and long-term time trends as well as the appropriate number of lagged observations for the dependent variable (see Methods). Table entries are coefficients (OLS standard errors) [Newey-West standard errors].

^{a} Coverage of Russia-Mueller on day t.

^{b} Average of the standardized values of coverage of NYT and ABC.

^{o} Lagged predictor is shown only when of interest.

Table S9: Predicting diversionary tweets (“Jobs”; J) from threatening media coverage (Russia-Mueller) and predicting suppression from diversionary tweets.

Dependent variable:	J tweets$_t$			NYT^a	ABC^a	Average^b
	(1)	(2)	(3)	(4)	(5)	(6)
NYT Russia/Mueller$_t$	0.005					
	(0.003) $p = .168$					
	[0.003] $p = .136$					
ABC Russia/Mueller$_t$		0.161				
		(0.062) $p = .010$				
		[0.057] $p = .005$				
Average Russia/Mueller$_t$			0.133			
			(0.051) $p = .009$			
			[0.046] $p = .004$			
J tweets$_{t-1}$	^{o}	^{o}	^{o}	-0.411	-0.051	-0.066
				(0.299) $p = .169$	(0.028) $p = .072$	(0.031) $p = .032$
				[0.280] $p = .142$	[0.026] $p = .052$	[0.026] $p = .012$
N	721	721	721	703	710	703

Notes.

Each model included control variables for each week during the sampling period and long-term time trends as well as the appropriate number of lagged observations for the dependent variable (see Methods). Table entries are coefficients (OLS standard errors) [Newey-West standard errors].

[a] Coverage of Russia-Mueller on day t.

[b] Average of the standardized values of coverage of NYT and ABC.

[o] Lagged predictor is shown only when of interest.

Table S10: Predicting diversionary tweets (“China”, “immigration”; CI) from threatening media coverage (Russia-Mueller) and predicting suppression from diversionary tweets.

Dependent variable:	CI tweets$_t$			NYT^a	ABC^a	Average^b
	(1)	(2)	(3)	(4)	(5)	(6)
NYT Russia/Mueller$_t$	0.009					
	(0.003) $p = .003$					
	[0.003] $p = .003$					
ABC Russia/Mueller$_t$		0.021				
		(0.052) $p = .684$				
		[0.049] $p = .668$				
Average Russia/Mueller$_t$			0.081			
			(0.040) $p = .044$			
			[0.038] $p = .031$			
CI tweets$_{t-1}$	^{o}	^{o}	^{o}	-0.358	-0.025	-0.031
				(0.293) $p = .221$	(0.025) $p = .325$	(0.026) $p = .234$
				[0.277] $p = .196$	[0.031] $p = .422$	[0.034] $p = .350$
N	721	721	721	703	710	703

Notes.

Each model included control variables for each week during the sampling period and long-term time trends as well as the appropriate number of lagged observations for the dependent variable (see Methods). Table entries are coefficients (OLS standard errors) [Newey-West standard errors].

[a] Coverage of Russia-Mueller on day t.

[b] Average of the standardized values of coverage of NYT and ABC.

[o] Lagged predictor is shown only when of interest.

Table S11: Predicting diversionary tweets (“China”; C) from threatening media coverage (Russia-Mueller) and predicting suppression from diversionary tweets.

Dependent variable:	C tweets$_t$			NYT^a	ABC^a	Average^b
	(1)	(2)	(3)	(4)	(5)	(6)
NYT Russia/Mueller$_t$	0.005					
	(0.002) $p = .028$					
	[0.003] $p = .030$					
ABC Russia/Mueller$_t$		0.026				
		(0.045) $p = .567$				
		[0.044] $p = .562$				
Average Russia/Mueller$_t$			0.059			
			(0.034) $p = .083$			
			[0.033] $p = .073$			
C tweets$_{t-1}$	^{o}	^{o}	^{o}	-0.583	-0.051	-0.067
				(0.342) $p = .089$	(0.031) $p = .106$	(0.030) $p = .027$
				[0.289] $p = .044$	[0.037] $p = .166$	[0.032] $p = .037$
N	721	721	721	703	710	703

Notes.

Each model included control variables for each week during the sampling period and long-term time trends as well as the appropriate number of lagged observations for the dependent variable (see Methods). Table entries are coefficients (OLS standard errors) [Newey-West standard errors].

[a] Coverage of Russia-Mueller on day t.

[b] Average of the standardized values of coverage of NYT and ABC.

[o] Lagged predictor is shown only when of interest.

Table S12: Predicting diversionary tweets ("Immigration"; I) from threatening media coverage (Russia-Mueller) and predicting suppression from diversionary tweets.

Dependent variable:		I tweets$_t$		NYT^a	ABC^a	Average^b
	(1)	(2)	(3)	(4)	(5)	(6)
NYT Russia/Mueller$_t$	0.004					
	(0.002) $p = .018$					
	[0.002] $p = .023$					
ABC Russia/Mueller$_t$		-0.004				
		(0.026) $p = .876$				
		[0.023] $p = .864$				
Average Russia/Mueller$_t$			0.032			
			(0.024) $p = .173$			
			[0.022] $p = .136$			
I tweets$_{t-1}$	^{o}	^{o}	^{o}	0.166	0.035	0.053
				(0.510) $p = .745$	(0.041) $p = .384$	(0.045) $p = .243$
				[0.547] $p = .762$	[0.043] $p = .413$	[0.058] $p = .360$
N	721	721	721	703	710	703

Notes.

Each model included control variables for each week during the sampling period and long-term time trends as well as the appropriate number of lagged observations for the dependent variable (see Methods). Table entries are coefficients (OLS standard errors) [Newey-West standard errors].

^{a} Coverage of Russia-Mueller on day t.

^{b} Average of the standardized values of coverage of NYT and ABC.

^{o} Lagged predictor is shown only when of interest.

Table S13: Predicting diversionary tweets (“China”, “jobs”, and “immigration”; CJI) from threatening media coverage (Russia-Mueller) and predicting suppression from diversionary tweets using a negative binomial regression model.

Dependent variable:		CJI tweets_t		NYT^a	ABC^a	Average^b
	(1)	(2)	(3)	(4)	(5)	(6)
NYT Russia/Mueller_t	0.019					
	(0.007) $p = .005$					
	[0.006] $p = .003$					
ABC Russia/Mueller_t		0.342				
		(0.104) $p = .001$				
		[0.101] $p = .001$				
Average Russia/Mueller_t			0.349			
			(0.090) $p = .000$			
			[0.084] $p = .000$			
CJI tweets_{t-1}	^{o}	^{o}	^{o}	-0.015	-0.081	
				(0.010) $p = .130$	(0.047) $p = .089$	
				[0.010] $p = .117$	[0.045] $p = .071$	
N	721	721	721	703	710	

Notes.

Each model included control variables for each week during the sampling period and long-term time trends as well as the appropriate number of lagged observations for the dependent variable (see Methods). Table entries are coefficients (standard errors) [Newey-West standard errors]. The suppression model for ABC (column 5) did not converge.

^{a} Coverage of Russia-Mueller on day t.

^{b} Suppression model for Average not estimable because average media coverage is not counted data.

^{o} Lagged predictor is shown only when of interest.

Table S14: Number of word pairs (percent) in bottom-right quadrants of panels in Figure 4, which occurred significantly more often in response to Russia/Mueller coverage in NYT or ABC News or average of standardized coverage of both, and which were associated with significant suppression of coverage the next day.

Media outlet	OLS	3SLS-Y2T[a]	3SLS-T2T[b]
NYT	16 (1.1)	74 (5.0)	24 (1.6)
ABC	23 (1.5)	14 (0.9)	57 (3.8)
Average[c]	62 (4.2)	87 (5.9)	35 (2.4)

Notes.

[a] 3SLS model where today's suppression is predicted from yesterday's tweets.

[b] 3SLS model where tomorrow's suppression is predicted from today's tweets.

[c] Average of the standardized coverage of NYT and ABC.

Table S15: Word pairs in tweets that occurred significantly more often in response to Russia/Mueller coverage in NYT and ABC News (average of standardized coverage) and that were associated with significant suppression the next day, in decreasing order of effect sizes.

	Word 1	Word 2	Divert t^a	Suppress t^b
1	tax	job	3.28	-3.57
2	job	china	2.56	-4.10
3	job	korea	2.90	-3.72
4	job	north	2.87	-3.68
5	jobs	tax	3.62	-2.61
6	job	made	2.61	-3.53
7	jobs	china	3.01	-3.12
8	today	job	2.93	-3.15
9	tax	china	3.22	-2.85
10	job	dems	2.25	-3.82
11	jobs	korea	3.42	-2.60
12	united	job	2.77	-3.18
13	media	job	2.13	-3.80
14	house	job	2.93	-2.93
15	jobs	north	3.35	-2.49
16	states	job	2.50	-3.25
17	job	republicans	2.12	-3.61
18	job	crime	2.68	-3.01
19	job	hunt	3.44	-2.16
20	good	job	2.44	-3.14
21	tax	korea	3.41	-2.12
22	media	tax	2.76	-2.72
23	job	long	2.42	-3.05
24	job	year	2.18	-3.28
25	jobs	job	2.48	-2.97
26	job	witch	3.41	-2.04
27	election	job	2.62	-2.81
28	america	job	2.10	-3.31
29	back	job	2.80	-2.57
30	tax	made	3.31	-2.02
31	tax	north	3.29	-1.97
32	media	jobs	2.36	-2.87
33	jobs	dems	2.49	-2.75
34	jobs	made	2.80	-2.41
35	jobs	united	3.07	-2.11
36	trump	job	2.16	-3.02
37	job	honor	2.95	-2.21
38	job	world	2.45	-2.70
39	great	job	2.96	-2.16
40	jobs	republicans	2.57	-2.54
41	big	jobs	2.12	-2.84
42	good	jobs	2.86	-2.08
43	jobs	states	2.72	-2.22
44	make	jobs	2.13	-2.80
45	job	working	2.03	-2.88
46	tax	dems	2.57	-2.31
47	years	job	2.10	-2.75
48	made	china	2.45	-2.38
49	tax	republicans	2.68	-2.13
50	president	job	2.79	-1.99
51	jobs	security	2.05	-2.72
52	job	hard	2.19	-2.57
53	jobs	long	2.72	-2.01
54	north	china	2.32	-2.23
55	make	tax	2.17	-2.37
56	jobs	year	2.23	-2.28
57	big	tax	2.00	-2.51
58	america	jobs	2.23	-2.28
59	korea	china	2.13	-2.28
60	tax	year	2.37	-2.01
61	people	job	2.02	-2.26
62	states	china	2.26	-2.02

Notes. ^{a,b} t-values of independent OLS models for diversion and suppression, respectively.
