# Supplementary material for: Using the president’s tweets to understand political diversion in the age of social media
Source: Nat Commun. 2020 Nov 10;11:5764. doi: 10.1038/s41467-020-19644-6 (PMC7655817; doi:10.1038/s41467-020-19644-6)
Supplement: Supplementary file 3 — Reporting Summary [file 41467_2020_19644_MOESM3_ESM.pdf]

nature research

Corresponding author(s): Lewandowsky

Last updated by author(s): Sep 19, 2020

Reporting Summary

Nature Research wishes to improve the reproducibility of the work that we publish. This form provides structure for consistency and transparency in reporting. For further information on Nature Research policies, see Authors & Referees and the Editorial Policy Checklist.

Statistics

For all statistical analyses, confirm that the following items are present in the figure legend, table legend, main text, or Methods section.

n/a	Confirmed	
☐	☒	The exact sample size (*n*) for each experimental group/condition, given as a discrete number and unit of measurement
☐	☒	A statement on whether measurements were taken from distinct samples or whether the same sample was measured repeatedly
☐	☒	The statistical test(s) used AND whether they are one- or two-sided<br>*Only common tests should be described solely by name; describe more complex techniques in the Methods section.*
☐	☒	A description of all covariates tested
☐	☒	A description of any assumptions or corrections, such as tests of normality and adjustment for multiple comparisons
☐	☒	A full description of the statistical parameters including central tendency (e.g. means) or other basic estimates (e.g. regression coefficient) AND variation (e.g. standard deviation) or associated estimates of uncertainty (e.g. confidence intervals)
☐	☒	For null hypothesis testing, the test statistic (e.g. F, t, r) with confidence intervals, effect sizes, degrees of freedom and P value noted<br>*Give P values as exact values whenever suitable.*
☒	☐	For Bayesian analysis, information on the choice of priors and Markov chain Monte Carlo settings
☐	☒	For hierarchical and complex designs, identification of the appropriate level for tests and full reporting of outcomes
☐	☒	Estimates of effect sizes (e.g. Cohen's *d*, Pearson's *r*), indicating how they were calculated

Our web collection on statistics for biologists contains articles on many of the points above.

Software and code

Policy information about availability of computer code

Data collection	see Methods
Data analysis	All analyses were done in R and/or Stata, specific packages are mentioned in the paper. R version 3.6.3; Stata IC version 16 (64-bit); R package MASS version 7.3-51.5; R package tm version 0.7-7; R package Rstata version 1.1.1.

For manuscripts utilizing custom algorithms or software that are central to the research but not yet described in published literature, software must be made available to editors/reviewers. We strongly encourage code deposition in a community repository (e.g. GitHub). See the Nature Research guidelines for submitting code & software for further information.

Data

Policy information about availability of data

All manuscripts must include a data availability statement. This statement should provide the following information, where applicable:

- Accession codes, unique identifiers, or web links for publicly available datasets
- A list of figures that have associated raw data
- A description of any restrictions on data availability

As specified in the Data Availability and Code Availability sections, all data and source code for analysis is available at https://osf.io/f9bqx/. There are no restrictions on access.

Field-specific reporting

Please select the one below that is the best fit for your research. If you are not sure, read the appropriate sections before making your selection.

☐ Life sciences ☒ Behavioural & social sciences ☐ Ecological, evolutionary & environmental sciences

For a reference copy of the document with all sections, see nature.com/documents/nr-reporting-summary-flat.pdf

Behavioural & social sciences study design

All studies must disclose on these points even when the disclosure is negative.

Study description	By analyzing President Trump's tweets and data from two media sources, the authors provide evidence suggesting that when the media reports on a topic potentially harmful to the president, he tweets about unrelated issues. Further evidence from this case study suggests that these diversionary tweets may also successfully reduce subsequent media coverage of the harmful topic.
Research sample	All of Donald Trump's tweets during the first 2 years of his presidency, all of New York Times coverage and ABC Evening News headlines during the same time period. The sampling period was chosen because it brackets the major event examined here, namely the effects of adverse media coverage relating to the Mueller investigation on President Trump's tweeting behaviour and the reciprocal effects of that on subsequent media coverage.
Sampling strategy	Exhaustive for the sources and sampling period just mentioned. We chose the newspaper of record (New York Times) and the corresponding TV News show (ABC Headline News). See paper for more details.
Data collection	Internet scraping and download of archives using a computer connected to the Internet (see Methods).
Timing	Data were collected after the first two years of Donald Trump's presidency, i.e. February 2019 onward.
Data exclusions	No data were excluded. All tweets and media materials available during the sampling period were used.
Non-participation	There were no human participants
Randomization	There was no random assignment of participants (no participants). Randomization was used only to create a null distribution for tweets.

Reporting for specific materials, systems and methods

We require information from authors about some types of materials, experimental systems and methods used in many studies. Here, indicate whether each material, system or method listed is relevant to your study. If you are not sure if a list item applies to your research, read the appropriate section before selecting a response.

Materials & experimental systems

n/a	Involved in the study	
☒	☐	Antibodies
☒	☐	Eukaryotic cell lines
☒	☐	Palaeontology
☒	☐	Animals and other organisms
☒	☐	Human research participants
☒	☐	Clinical data

Methods

n/a	Involved in the study	
☒	☐	ChIP-seq
☒	☐	Flow cytometry
☒	☐	MRI-based neuroimaging
